# Validation of GLIM criteria for hospital malnutrition diagnosis by comparison of three different anthropometric approaches to evaluate reduced muscle mass: a prospective cohort study

**DOI:** 10.3389/fnut.2024.1438158

**Published:** 2024-12-05

**Authors:** Mostafa Shahraki Jazinaki, Mohammad Safarian, Seyyed Mostafa Arabi, Jamshid Jamali, Abdolreza Norouzy

**Affiliations:** ^1^Student Research Committee, Mashhad University of Medical Sciences, Mashhad, Iran; ^2^Department of Nutrition, Faculty of Medicine, Mashhad University of Medical Sciences, Mashhad, Iran; ^3^Metabolic Syndrome Research Centre, Mashhad University of Medical Sciences, Mashhad, Iran; ^4^Noncommunicable Diseases Research Center, Neyshabur University of Medical Sciences, Neyshabur, Iran; ^5^Healthy Ageing Research Centre, Neyshabur University of Medical Sciences, Neyshabur, Iran; ^6^Social Determinants of Health Research Center, Mashhad University of Medical Sciences, Mashhad, Iran; ^7^Department of Biostatistics, School of Health, Mashhad University of Medical Sciences, Mashhad, Iran

**Keywords:** validation, The Global Leadership Initiative on Malnutrition, nutritional assessment, malnutrition, clinical outcomes

## Abstract

**Background and aim:**

The Global Leadership Initiative on Malnutrition (GLIM) recently proposed a new malnutrition diagnostic tool known as the GLIM criteria. The GLIM criteria need confirmed validation before being widely used in each population or healthcare system. This study aimed to investigate the validation of the GLIM criteria for malnutrition diagnosis in hospitalized patients.

**Methods:**

The content validity was assessed by calculating the content validity ratio (CVR) and content validity index (CVI). Subjective global assessment (SGA) is considered the reference tool to diagnose malnutrition in concurrent validation. In addition, the Kuder–Richardson 20 was used to evaluate the reliability of the GLIM criteria. Furthermore, hospital mortality, length of hospitalization (LOS), prolonged hospital stays (LOS >6 days), 30-day hospital readmission, and 30- and 60-day mortality were identified as malnutrition-related outcomes in predictive validity.

**Results:**

A total of 332 adult/elderly hospitalized patients (median age: 58 (IQR: 24.7), 60.5% men) were enrolled to present the study. Appling GLIM criteria by considering the calf circumference < 31 cm in both genders or mid-upper arm (MUAC) < 23 cm in men and MUAC <22 cm in women as reduced muscle mass had an appropriate accuracy (84.6 and 83.4%, respectively), good ability to distinguish malnourished patients (AUC ROC: 0.85 and 0.83, respectively), satisfactory sensitivity (89.58 and 84.02%, respectively), and satisfactory specificity (81 and 83%, respectively) compared to the SGA tool. Furthermore, the reliability of the GLIM criteria for malnutrition diagnosis in hospitalized patients was acceptable in all 3 applied approaches (KR-20 > 0.5). The malnutrition diagnosed by GLIM criteria could significantly predict the odds of prolonged hospital stays, 30-day hospital readmission, and 60-day mortality, while it had no significant association with the risk of hospital mortality.

**Conclusion:**

The current study revealed that applying GLIM criteria had satisfactory validity in diagnosing hospital malnutrition in non-critically ill hospitalized patients.

## 1 Introduction

Malnutrition has been recognized as an independent predictor of adverse clinical outcomes in hospitalized patients, including prolonged hospital stays, morbidity, infection, and mortality (1, 2). The primary causes of malnutrition include dramatic reductions in food intake, malabsorption, and the stress brought on by inflammatory processes, which lead to changes in body composition and decreased function (3–6). The subjective global assessment (SGA) is one of the common standard tools for diagnosing malnutrition and determining its severity (7). SGA diagnoses malnutrition as a low-cost, simple, and non-invasive method at the patient’s bedside by subjectively examining changes in body composition, food intake, and body function (8). While SGA is a nutritional assessment tool that can usually predict prolonged hospital stays, readmissions, postoperative complications, and mortality, its effectiveness is very dependent on the evaluator’s expertise and the patients’ recollection (9, 10). International guideline committees have recently decided to classify the types of malnutrition according to their etiologic basis into four groups: (a) chronic disease with minimal or no perceived inflammation; (b) chronic disease or conditions with sustained inflammation; (c) acute disease or injury with severe inflammation; and (d) pure chronic starvation not related to the disease (3, 11).

In the last decade, clinical nutrition researchers have sought to introduce new criteria and terminology that could be used globally in all medical settings for diagnosing malnutrition (12). The Global Leadership Initiative on Malnutrition (GLIM) in 2018 introduced evidence-based operational criteria that are known as the “GLIM criteria” for diagnosing the mentioned types of protein-energy malnutrition. These criteria comprise three phenotypic criteria (reduced muscle mass, low body mass index, and weight loss) and two etiological criteria (reduced food intake or assimilation, and inflammation). By providing at least one etiological and one phenotypic criterion, the diagnosis of malnutrition is made for the patients (the details of the evaluation of each criterion are provided in Supplementary Table S1) (3, 11, 12). GLIM criteria as an operational tool for diagnosing malnutrition as a consensus-based tool must be validated in populations before being widely disseminated and used (12). The significant association between malnutrition diagnosed by using GLIM criteria and poor prognosis was demonstrated in several populations, such as individuals with cardiovascular disease (13), tumors (14), and patients admitted to the emergency ward (15). To the best of our knowledge, the validation of the GLIM tool has not been assessed yet in Iranian non-critically ill hospitalized patients. In addition, due to the limited prospective validation studies for the performance of the GLIM tool in hospitalized patients, contradictory findings regarding the GLIM criteria’s ability to predict clinical outcomes (16–19), and assessment of content validity, and reliability evaluations in the limited number of previous studies, the present study aims to evaluate the validation of the GLIM malnutrition diagnostic criteria’s performance in the population of Iranian non-critically ill hospitalized adults/elderly patients in comparison with SGA as the reference diagnostic tool.

## 2 Materials and methods

### 2.1 Study design

This multicenter, prospective cohort, observational study was conducted in Iran’s Mashhad City’s two major hospitals (Quaem and Imam Reza hospitals) between March and November 2023. The current study was approved by the Mashhad University of Medical Science Ethics Committee (Serial number: IR.MUMS.MEDICAL.REC.1401.681). In addition, all participants completed and signed the informed consent form before being involved in the study.

### 2.2 Study population

Patients included in the study were from all wards (except critically ill patients) of Mashhad two large hospitals (Ghaem and Imam Reza hospitals) and both sexes. The inclusion criteria of the present study include the following: (a) adults (age ≥ 18); (b) Lucid-oriented patients or the presence of family members with accurate information from patients; and (c) there were enough data from patients in the hospital files and information systems.

The exclusion criteria of the present study include the following: (a) patients with amputations of upper and lower limbs (superior and/or inferior) for whom anthropometric measurements were not possible; (b) non-orientated patients without informed companions; and (c) pregnant or lactating mothers.

### 2.3 Content validity

To evaluate content validity, the panel of experts (including 16 individuals with PhDs/MDs, PhDs, and PhD candidates in nutrition) was surveyed regarding the degree of necessity (with the calculation of CVR), relevance, and clarity (with the calculation of CVI relevancy).

### 2.4 Reliability assessment

The reliability of the GLIM malnutrition diagnosis criteria was evaluated by calculating the Kuder–Richardson 20 (KR20) (Cronbach’s alpha). KR20 > 0.50 was identified as acceptable reliability (20).

### 2.5 Data collection

Based on the GLIM validation guidance, in the first 48 h of hospital admission, patients were involved in the study, and requirement assessments were performed by trained researchers. Before going beside the patient’s bed, information including reason for admission (chief complaints), past medical history (PMH), drug consumption list, demographic information such as name, age, gender, and laboratory data such as C-reactive protein (CRP) levels for the past 48 h was noted from the patient’s medical record file and hospital information system (HIS). The Charlson Comorbidity Index (CCI) was calculated based on the co-morbidities of each patient, indicating the severity of the conditions and the probability of survival in the next 10 years (21). First, the patient was asked about symptoms affecting food intake in the past 2 weeks in the form of a checklist, such as pain while eating, nausea, vomiting, diarrhea, constipation, dental problems, and anorexia. The patient’s food intake was recorded using a 24-h recall, and calories were estimated by calculating the number of units received and based on the amount of energy in each unit of food groups.

The energy requirement of patients was estimated using the weight-based equations (22–24).

By following the formula: energy intake/energy requirements ×100, the percentage of energy balance was estimated, and then, the patient was asked what percentage of his current intake was in the past 2 weeks (100, 75, 50, 25%, or 0%). The energy intake of patients receiving enteral nutrition in the last 2 weeks was calculated based on the volume received and the type of product consumed. The presence of inflammatory conditions in patients was identified when CRP-reactive protein levels were more than 5 mg/L. If the CRP levels of the last 48 h of the patient were not available, the inflammatory conditions of the patient’s body were interpreted based on the instructions introduced in the GLIM validation guidance (12).

The patient’s weight was measured using a Seka scale available in the nursing station with an accuracy of 0.1 kg. Patients were placed on the scale with minimal light clothing and no shoes, and then, their weight was recorded. The patient was asked about his usual weight in the last 6 months and 1 year, respectively. Then, by following the formula, the percent of weight loss was calculated: ((Usual weight-current weight)/current weight) × 100. If the patient was unable to walk, the patient’s weight changes were recorded as a self-report, and if the patient was not oriented, the companion who had complete information about the patient was asked whether the patient had lost more than 5% weight in the last 6 months. Or has it decreased by more than 10% in the last 12 months or not?

The height of the patient was measured by using a stadiometer located at the nursing station in a situation where the patient was without shoes, heels against the backboard, standing with arms down, feet together, knees straight, and face forward (the Frankfurt horizontal plane) with an accuracy of 0.01 M. If the patient was unable to move, the height was reported by self-report, and in cases of lack of knowledge, it was estimated by measuring the length of the ulna (25). Body mass index was calculated by dividing weight (kg) by the square of height (m^2^) (weight (kg)/ height^2^ (m^2^)). In patients aged >70 years, BMI < 22 kg/m^2^, and for patients aged <70 years, BMI < 20 kg/m^2^ was considered as low BMI. To evaluate the reduced muscle mass, two separate anthropometric measurement methods were considered, including the calf circumference (CC) and the mid-upper arm circumference (MUAC). To measure CC, the maximum calf circumference of the patient in the condition that the leg had an angle of 90 degrees to the ground was measured using a flexible non-stretch tape. To determine the reduced muscle mass based on calf circumference, two cutoff points were applied: (a) CC ≤ 34 cm in men and CC ≤33 cm in women; (b) CC < 31 cm in both genders. Mid-upper arm circumference (MUAC) was measured by measuring the midpoint between the olecranon and acromion using flexible non-stretch tape. In addition, for identifying reduced muscle mass based on MUAC, MUAC <23 cm in men and MUAC <22 cm in women were determined as the cutoff points (26). The meeting of at least one etiological criterion and one phenotypic criterion led to the diagnosis of malnutrition. However, the determination of malnutrition severity depended on just phenotypic criteria (Supplementary Table S1) (3). In the current study, SGA was used according to the approach introduced by Detsky et al. (27). Assessment of muscle mass loss, subcutaneous fat loss, fluid accumulation, unwanted weight loss, reduced food intake, and decreased ability to perform and function were the main components of the SGA tool. The anatomical regions, including temporal (for the non-elderlies), pectoral, deltoid (supraclavicular and infraclavicular areas), quadriceps, and gastrocnemius, were examined for muscle mass loss. Furthermore, the orbital, triceps, and area covering the ribs were examined for subcutaneous fat loss. There were three categories for the severity of muscle mass loss and subcutaneous fat loss: absent, mild/moderate, and severe. Using the SGA tool, the nutritional status of the patients was subjectively classified into three levels: (A) well-nourished, (B) mild-to-moderate malnutrition, and (C) severe malnutrition.

Some clinical outcomes, such as hospital mortality, length of hospital stay, and prolonged hospital stays, were collected using the hospital information system, while data about some other outcomes, such as 30-day readmission to the hospital and 30-day and 60-day mortality, were collected using the contact information that was collected from the patients.

### 2.6 Statistical analyses

The sample size was calculated based on the nutrition prevalence of 23.92% reported by Poudineh et al. (28), an expected kappa of 0.648 (16), a minimum acceptable kappa of 80%, with 90% power, a statistically significance level of 0.05 (two-tailed), and an anticipated dropout rate of 20%. Therefore, the estimated sample size was 282 participants.

Categorical variables were reported as absolute (N) and percentage (%), and the chi-square test was performed to compare well-nourished and malnourished groups. Continuous variables with a normal distribution were expressed as mean ± standard, and Student’s *t*-test was used to compare them between the two groups. Furthermore, continuous variables with a non-normal distribution were expressed as median (first-to-third interquartile range), and to compare them between the two groups, the Mann–Whitney test was performed. The normal distribution of quantitative variables was checked with the Kolmogorov–Smirnov test. The SGA tool was identified as the reference tool to evaluate the concurrent validity of the GLIM criteria. By using the kappa coefficient (k), the degree of agreement between the GLIM criteria and SGA for malnutrition diagnosis was evaluated. This value was divided into five categories: 1.00 is considered perfect, 0.81–0.99 as almost perfect, 0.61–0.80 as substantial, 0.41–0.60 as moderate, 0.21–0.40 as fair, and ≤ 0.20 as poor agreement (29). Furthermore, the accuracy, specificity, sensitivity, positive and negative predictive values, and area under the receiver operating characteristic (ROC) curve with a confidence interval (CI) of 95% were calculated to investigate the concurrent validity of GLIM criteria compared to SGA. To determine the concurrent validity as satisfactory, both sensitivity and specificity values had to be 80% < (3). The ROC AUC value that indicates the GLIM ability to distinguish malnourished patients is interpreted as follows: > 0.9 as excellent, 0.8–0.9 as good, 0.7–0.8 as poor, 0.6–0.7 as worthless, and 0.5–0.6 as failed (30). The predictive validity of the GLIM criteria was evaluated using logistic regression, which considered a prolonged length of stay (the length of stay in the hospital is greater than the median value of LOS = 6 days), 30-day hospital readmission, 30-day mortality, and 60-day mortality as independent variables, and Cox regression which considered in-hospital mortality as an independent variable were performed. Also, multivariate analysis was carry out to adjust the influence of confounders on the results. All analyses performed in this study were conducted using IBM SPSS Statistics version 27.0 (IBM Corporation, SPSS, INC., Chicago, IL, United States). *p*-values of <0.05 were interpreted as statistical significance in all tests.

## 3 Results

### 3.1 The content validity

The content validity of all five GLIM Malnutrition Diagnostic Tool criteria was confirmed by calculating CVR, CVI relevancy, and CVI clarity based on the experts’ opinions (the details of CVI and CVR scores for each criterion are provided in Supplementary Table S2).

### 3.2 General characteristics of participants

**Figure 1:**
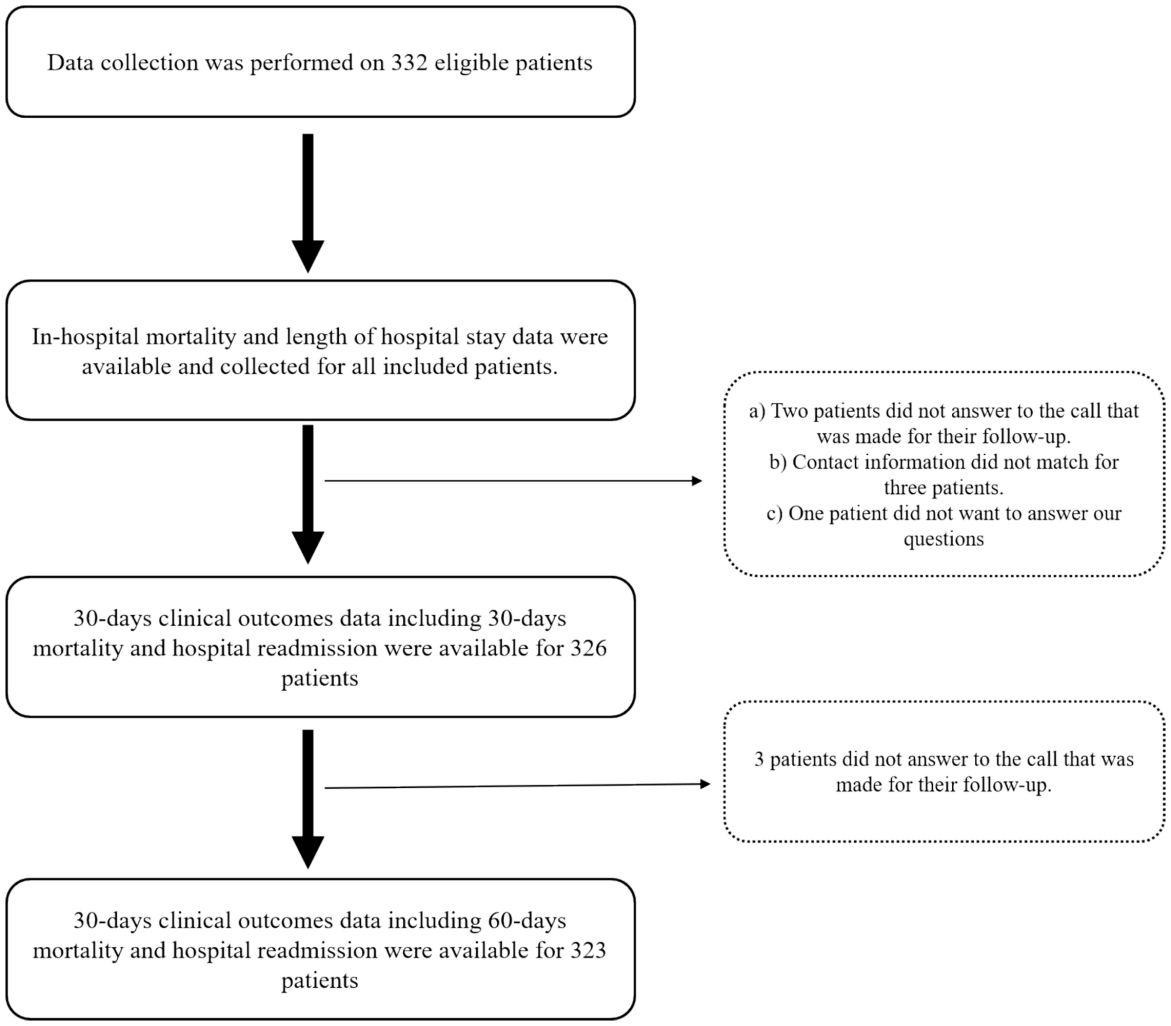
Flow chart of study design and data collection.

As shown in Figure 1, a total of 332 hospitalized patients were eligible to be included in this study. The median age of the patients was 58 years; 60.5% of them were men, and 32.2% of them were older than 65. The main complaints leading to hospitalization of patients were hematological (n = 99, 22.8%), gastrointestinal (n = 82, 22.7%), neurological (n = 23, 6.9%), cardiac (n = 22, 6.6%), and nephrological and pulmonary (n = 7, 2.1%). Furthermore, cancer (n = 86, 25.9%), hypertension (n = 52, 15.7%), surgery (n = 50, 15.1%), diabetes (n = 48, 14.5%), CKD (n = 16, 4.8%), and CVA (n = 14, 4.2%) were the most common PMHs of the participants. The median CCI of the patients was 3, serum CRP levels were available for 153 patients, and their median levels were 18.6 mg/L. The median length of stay of patients in the hospital was 6 days, and the length of hospital stays was longer than 6 days in 47.6% of patients, which was considered a prolonged hospital stay. Using the SGA tool for diagnosing malnutrition led to the identification of 144 (43.4%) patients as malnourished. The severity of malnutrition based on the SGA approach was determined for 75 (22.6%) patients as moderate and 69 (20.8%) patients as severe (Supplementary Table S3). The prevalence of hospital mortality among the included patients was 6.0%. Data on 30-day hospital readmission and 30-day mortality were available for 326, and data on 60-day mortality were available for 323 participants. The prevalence of 30-day hospital readmission, 30-day mortality, and 60-day mortality among the included participants was 25.5, 10.4, and 20.1%, respectively (Table 1).

**Table 1:** Characteristics of hospitalized patients categorized by malnutrition diagnosis using GLIM criteria.

		Using CC ≤ 34 for men and CC ≤ 33 for women to evaluate reduced muscle mass			Using MUAC < 23 for men and MUAC < 22 for women to evaluate reduced muscle mass			Using CC < 31 for both genders to evaluate reduced muscle mass		
Variables	All sample	Well-nourished	Malnourished	*p*-Value	Well-nourished	Malnourished	*p*-Value	Well-nourished	Malnourished	*p*-Value
		121 (36.4%)	211 (63.6%)		179 (53.9%)	153 (46.1%)		167 (50.3%)	165 (49.7%)	
Age (years)	58.0 (43.0, 68.0)	54.0 (42.0, 65.0)	61.0 (45.0, 69.0)	**0.01** ^b^	56.0 (41.0, 66.0)	61.0 (46.0, 71.0)	0.06^b^	55.0 (41.0, 65.0)	61.0 (46.5, 71.0)	**0.02** ^b^
Age > 65 years	107 (32.2%)	31 (25.6%)	76 (36.0%)	0.05^a^	50 (27.9%)	57 (37.3%)	0.07^a^	43 (25.7%)	64 (38.8%)	**0.01** ^a^
Men	201 (60.5%)	67 (55.4%)	134 (63.5%)	0.14^a^	110 (61.5%)	91 (59.5%)	0.71a	100 (59.9%)	101 (61.2%)	0.80^a^
CCI	3.0 (1.0, 4.0)	2.0 (0.0, 3.0)	3.0 (2.0, 4.0)	**<0.001** ^b^	2.0 (1.0, 4.0)	3.0 (2.0, 4.0)	**0.004** ^b^	2.0 (1.0, 3.0)	3.0 (2.0, 4.5)	**<0.001** ^a^
Past medical history										
Cancer	86 (25.9%)	17 (14.0%)	69 (32.7%)	**<0.001** ^a^	35 (19.6%)	51 (33.3%)	**0.004** ^a^	31 (18.6%)	55 (33.3%)	**0.002** ^a^
HTN	52 (15.7%)	19 (15.7%)	33 (15.6%)	0.98^a^	32 (17.9%)	20 (13.1%)	0.23^a^	30 (18.0%)	22 (13.3%)	0.24^a^
Surgery	50 (15.1%)	19 (15.7%)	31 (14.7%)	0.80^a^	28 (15.6%)	22 (14.4%)	0.74^a^	23 (13.8%)	27 (16.4%)	0.50^a^
DM	48 (14.5%)	18 (14.9%)	30 (14.2%)	0.87^a^	31 (17.3%)	17 (11.1%)	0.10^a^	28 (16.8%)	20 (12.1%)	0.22^a^
CVA	14 (4.2%)	3 (2.5%)	11 (5.2%)	0.27^d^	4 (2.2%)	10 (6.5%)	0.05^d^	3 (1.8%)	11 (6.7%)	0.03^d^
CKD	16 (4.8%)	9 (7.4%)	7 (3.3%)	0.09^a^	12 (6.7%)	4 (2.6%)	0.12^d^	11 (6.6%)	5 (3.0%)	0.19^d^
Other PMH	94 (28.3%)	33 (27.3%)	61 (28.9%)	0.75^a^	50 (27.9%)	44 (28.8%)	0.86^a^	46 (27.5%)	48 (29.1%)	0.75^a^
Nutritional features										
Energy intake (kcal/day)	1004.0 (313.6, 1580.2)	1299.0 (799.5, 1724.0)	876.0 (192.2, 1406.0)	**<0.001** ^b^	1115.0 (540.0, 1641.5)	891.0 (205.2, 1370.7)	**0.03** ^a^	1178.0 (641.0, 1644.0)	850.0 (227.0, 1362.5)	**0.003** ^b^
Usual weight	70.0 (60.0, 80.0)	75.0 (67.0, 85.5)	65.0 (57.0, 74.0)	**<0.001** ^b^	72.0 (65.0, 83.0)	65.0 (55.0, 74.0)	**<0.001** ^b^	73.0 (65.0, 84.0)	65.0 (55.0, 73.0)	**<0.001** ^b^
Current weight	65.1 (55.2, 75.2)	76.2 ± 14.9	60.5 ± 12.1	**<0.001** ^c^	72.0 (65.0, 81.6)	56.3 (48.5, 65.0)	**<0.001** ^b^	72.7 (65.0, 82.0)	57.5 (50.2, 65.2)	**<0.001** ^b^
Height	1.68 (1.60, 1.75)	1.69 (1.61, 1.75)	1.66 (1.60, 1.73)	0.13^b^	1.67 ± 0.09	1.66 ± 0.09	0.33^c^	1.68 ± 0.09	1.66 ± 0.09	0.22^c^
BMI	23.6 ± 5.0	26.7 (24.1, 29.3)	21.5 (19.1, 24.3)	**<0.001** ^b^	25.7 (23.1, 29.0)	20.3 (17.9, 22.7)	**<0.001** ^b^	25.8 (23.4, 29.1)	20.6 (18.0, 22.9)	**<0.001** ^b^
CC	32.0 (29.5, 35.0)	35.0 (32.0, 37.4)	30.6 (28.0, 32.8)	**<0.001** ^b^	33.5 (31.0, 36.0)	30.2 (27.4, 32.0)	**<0.001** ^b^	34.0 (32.0, 36.0)	30.0 (27.0, 32.0)	**<0.001** ^b^
MUAC	25.6 (23.0, 28.9)	28.6 (26.3, 31.0)	24.0 (22.0, 26.4)	**<0.001** ^b^	28.0 (25.4, 30.0)	24.0 (21.2, 25.5)	**<0.001** ^b^	28.0 (25.5, 30.4)	24.0 (21.5, 25.6)	**<0.001** ^b^
GLIM criteria										
Phenotypic criteria (number)	1.0 (1.0, 2.0)	0.0 (0.0, 1.0)	2.0 (1.0, 3.0)	**<0.001** ^b^	0.0 (0.0, 0.0)	1.0 (1.0, 2.0)	**<0.001** ^b^	0.0 (0.0, 0.0)	2.0 (1.0, 3.0)	**<0.001** ^b^
	1.0 (0.0, 1.0)									
	1.0 (0.0, 2.0)									
Etiologic criteria (number)	1 (1, 2)	0 (0, 1)	1 (1, 2)	**<0.001** ^b^	1 (0, 1)	1 (1, 2)	**<0.001** ^b^	1 (0, 1)	1 (1, 2)	**<0.001** ^b^
Weight loss (%)	134 (40.4%)	12 (9.9%)	122 (57.8%)	**<0.001** ^a^	12 (6.7%)	122 (79.7%)	**<0.001** ^a^	13 (7.8%)	121 (73.3%)	**<0.001** ^a^
Low body mass index (kg/m^2^)	95 (28.6%)	10 (8.3%)	85 (40.3%)	**<0.001** ^a^	10 (5.6%)	85 (55.6%)	**<0.001** ^a^	10 (6.0%)	85 (51.5%)	**<0.001** ^a^
Reduced muscle mass	237 (71.4%)	45 (37.2%)	192 (91.0%)	**<0.001** ^a^	6 (3.4%)	56 (36.6%)	**<0.001** ^a^	18 (10.8%)	108 (65.5%)	**<0.001** ^a^
	62 (18.7%)									
	126 (38.0%)									
Reduced food intake or assimilation	114 (34.3%)	13 (10.7%)	101 (47.9%)	**<0.001** ^a^	30 (16.8%)	84 (54.9%)	**<0.001** ^a^	25 (15.0%)	89 (53.9%)	**<0.001** ^a^
Nutrition impact symptoms	150 (45.2%)	40 (33.1%)	110 (52.1%)	**<0.001** ^a^	61 (34.1%)	89 (58.2%)	**<0.001** ^a^	55 (32.9%)	95 (57.6%)	**<0.001** ^a^
Inflammation	255 (67.8%)	43 (35.5%)	182 (86.3%)	**<0.001** ^a^	98 (54.7%)	127 (83.0%)	**<0.001** ^a^	87 (52.1%)	138 (82.6%)	**<0.001** ^a^
Serum CRP levels (N = 153)	18.6 (4.4, 104.3)	3.9 (2.1, 15.8)	45.4 (8.3, 132.3)	**<0.001** ^b^	13.3 (2.8, 84.0)	31.9 (7.3, 123.2)	**0.02** ^b^	6.8 (2.5, 83.0)	35.4 (8.0, 126.6)	**0.001** ^b^
Clinical outcomes										
Hospital LOS (days)	6.0 (4.0, 9.0)	6.0 (4.0, 9.0)	7.0 (4.0, 10.0)	0.51^b^	6.0 (4.0, 9.0)	7.0 (4.0, 11.0)	0.17^a^	6.0 (4.0, 8.0)	7.0 (4.0, 11.5)	**0.02** ^b^
Prolong hospital stay (>6 days)	158 (47.6%)	52 (43.0%)	106 (50.2%)	0.20^a^	79 (44.1%)	79 (51.6%)	0.17^a^	69 (41.3%)	89 (53.9%)	**0.02** ^a^
Hospital mortality	20 (6.0%)	3 (2.5%)	13 (8.1%)	0.05^d^	7 (3.9%)	13 (8.5%)	0.08^a^	6 (3.6%)	14 (8.5%)	0.06^a^
30-day hospital readmission (N = 326)	83 (25.5%)	17 (14.2%)	66 (32.0%)	**<0.001** ^a^	35 (19.8%)	48 (32.2%)	**0.01** ^a^	34 (20.5%)	49 (30.6%)	**0.03** ^a^
30-day mortality (N = 326)	34 (10.4%)	6 (5.0%)	28 (13.6%)	**0.01** ^a^	13 (7.3%)	21 (14.1%)	**0.04** ^a^	12 (7.2%)	22 (13.8%)	0.05^a^
60-day mortality (N = 323)	65 (20.1%)	12 (10.1%)	53 (26.0%)	**<0.001** ^a^	23 (13.1%)	42 (28.6%)	**<0.001** ^a^	21 (12.7%)	44 (27.8%)	**<0.001** ^a^

CCI, Charlson Comorbidity Index; DM, diabetes mellitus; CVA, cerebral vascular accident; CKD, chronic kidney disease; PMH, past medical history; CC, calf circumference, MUAC, mid-upper arm circumference; GLIM, Global Leadership Initiative on Malnutrition; CRP, C-reactive protein; LOS, length of hospital stays. Statistical significance with a *p*-value < 0.05 is indicated in bold.

a Chi-square test.

b Mann–Whitney test.

c Student’s *t*-test.

d Fisher’s test.

The prevalence of the symptoms that affect food intake and nutritional status among the included patients was 45.2%. Furthermore, anorexia, nausea and vomiting, and pain when eating were the most common symptoms that affected food intake (the frequency of each of the symptoms affecting the patient’s food intake in the included patients is shown in Supplementary Table S4).

The height and presence of symptoms that affect food intake and reduced muscle mass (based on CC ≤ 34 for men and CC ≤ 33 for women) were significantly higher in men than in women, while the BMI and prevalence of hypertension were significantly higher in women than in men. However, in other variables, no significant difference was observed between the two genders (Supplementary Table S3).

### 3.3 Results of using GLIM malnutrition diagnostic criteria that use CC ≤ 34 cm for men and CC ≤33 cm for women to evaluate reduced muscle mass in hospitalized patients

Using this tool showed that 211 (63.6%) of the patients were malnourished. Malnutrition severity was moderate in 115 (34.6%) and severe in 96 (28.9%) of patients (Supplementary Table S3). Furthermore, in malnourished patients, the energy intake, usual weight, current weight, BMI, CC, and MUAC were significantly lower than in well-nourished patients. Age, CCI, number of individuals with a past medical history of cancer, serum CRP levels, presence of symptoms affecting nutrition status, number of etiological and phenotypic criteria that were met, and the meeting of each of the GLIM criteria were significantly higher in malnourished patients than others. In addition, 30-day readmission and 30- and 60-day mortality occurred significantly more in malnourished patients than in well-nourished patients. No significant difference was detected among other variables between the two groups of malnourished and well-nourished patients. Furthermore, reduced muscle mass and the presence of inflammation were the most common phenotypic and etiologic criteria among the patients with malnutrition diagnoses, respectively (Table 1).

### 3.4 Results of using GLIM malnutrition diagnostic criteria that use MUAC < 23 cm for men and MUAC < 22 cm for women to evaluate reduced muscle mass in hospitalized patients

Using this tool led to the diagnosis of 153 (46.1%) patients as malnourished. The severity of malnutrition was moderate for 57 (17.2%) and severe for 96 patients (28.9%) (Supplementary Table S3). The energy intake, usual and current weight, BMI, CC, and MUAC were significantly lower in malnourished patients than in others. Meanwhile, CCI, number of individuals with a past medical history of cancer, serum levels of CRP, the number of etiological and phenotypic criteria that were met, and the frequency of meetings for each of the five criteria were significantly higher in malnourished patients than in well-nourished patients. In addition, the occurrence of 30-day readmissions and 30- and 60-day mortality was significantly higher in malnourished patients than in others. However, there was no significant difference in other variables between malnourished and well-nourished patients. Significant weight loss and the presence of inflammation were the most prevalent phenotypic and etiologic criteria in the malnourished patients, respectively (Table 1).

### 3.5 Result of using GLIM malnutrition diagnostic criteria that uses CC < 31 cm for both genders to evaluate reduced muscle mass in hospitalized patients

The use of this tool demonstrated that 165 (49.7%) of the included patients were malnourished. The severity of malnutrition was determined to be moderate for 69 (20.8%) and severe for 96 (28.9%) of the included patients (Supplementary Table S3). The age, number of individuals with a past medical history of cancer, serum CRP levels, CCI, number of patients aged ≥65 years, number of phenotypic and etiologic criteria that met, and meeting each of the five GLIM criteria were significantly higher in the malnourished patients than in the well-nourished, while energy intake, usual and current weight, BMI, CC, and MUAC were significantly lower in malnourished patients than others. Furthermore, the length of hospital stays (LOS), prevalence of prolonged hospital stays (hospital LOS > 6 days), 30-day readmission, and 60-day mortality were significantly higher in malnourished patients than in well-nourished patients, while there was no significant difference in the other variables between the two groups. The most common phenotypic and etiologic criteria among the malnourished patients were significant weight loss and the presence of inflammation, respectively (Table 1).

### 3.6 Concurrent validity of GLIM criteria

Applying the CC ≤ 34 cm in men and the CC ≤ 33 cm in women as a cutoff point to evaluate reduced muscle mass in GLIM criteria led to this tool having a moderate agreement with the SGA tool (*κ* = 0.50, *p* < 0.001). Furthermore, the accuracy of this tool was 74.4%, and it had a fair ability to distinguish malnourished people (AUC ROC: 0.76). However, the specificity of this tool (60.00%), contrary to its sensitivity (93.75%), was not satisfactory compared to SGA. By choosing the MUAC <23 cm for men and the MUAC <22 cm for women to evaluate reduced muscle mass, the GLIM criteria and SGA had a substantial agreement (*κ* = 0.66, *p* < 0.001). Furthermore, the accuracy of this tool was 83.4%, its sensitivity (84.02%) and specificity (83%) were satisfactory, and the ability of GLIM criteria to distinguish malnourished patients was considered good (AUC ROC: 0.83), compared to SGA as the reference tool. Considering CC <31 cm in men and women as reduced muscle mass led to the GLIM malnutrition diagnostic criteria having a substantial agreement with SGA (κ = 0.69, p < 0.001), and its accuracy was 84.6%. Furthermore, this tool had a good ability to distinguish malnourished patients (AUC ROC: 0.85). In addition, the sensitivity (89.58%) and specificity (81.00%) of this tool were satisfactory compared to SGA (Table 2 and Supplementary Figure S1).

**Table 2:** Concurrent validity of GLIM criteria for malnutrition diagnosis considering subjective global assessment as a reference in hospitalized patients.

Statistical parameters of concurrent validity	Using CC ≤ 34 for men and CC ≤ 33 for women to evaluate low muscle mass	Using MUAC < 23 for men and MUAC < 22 for women to evaluate low muscle mass	Using CC < 31 for both genders to evaluate low muscle mass
Accuracy (%)	74.4	83.4	84.6
Kappa (*p*-value)	0.506 **<0.001**	0.665 **<0.001**	0.693 **<0.001**
AUC ROC (CI 95%)	0.767 (0.715, 0.818)	0.835 (0.789, 0.882)	0.852 (0.808, 0.896)
Sensitivity (%)	93.75	84.02	89.58
Specificity (%)	60.00	83.00	81.00
Positive predictive value (%)	63.98	79.08	78.18
Negative predictive value (%)	92.56	87.15	91.01

AUC, area under the curve; ROC, receiver operating characteristic, CI, confidence interval; GLIM, Global Leadership Initiative on Malnutrition; CC, calf circumference; MUAC, mid-upper arm circumference. Statistical significance with a *p*-value < 0.05 is indicated in bold.

### 3.7 Reliability of GLIM criteria

Kuder–Richardson index (Cronbach’s alpha) for GLIM criteria by considering CC ≤34 cm in men and CC ≤33 cm in women, MUAC <23 cm in men and MUAC <22 cm in women, and CC <31 cm for both genders to detect reduced muscle mass were 0.52, 0.55, and 0.57, respectively. Therefore, all three methods that were used had acceptable reliability.

### 3.8 Predictive validity of GLIM criteria

As shown in Table 1, no significant difference was detected between malnourished and well-nourished patients in the frequency of hospital mortality. However, 30-day hospital readmission and 60-day mortality were significantly higher in malnourished patients than in well-nourished patients, regardless of the type of cutoff points and methods used to assess reduced muscle mass. The hospital LOS and prevalence of prolonged hospital stay (>6 days) were significantly higher in malnourished patients than in well-nourished patients diagnosed by using the GLIM malnutrition diagnostic criteria that uses CC < 31 cm in both genders as a cutoff point to evaluate reduced muscle mass, while no significant difference was detected in the other methods. In addition, 30-day mortality was significantly higher in malnourished patients than well-nourished patients diagnosed by GLIM criteria when using CC ≤ 34 cm in men and CC ≤ 33 cm in women or when considering MUAC<23 cm in men and MUAC<22 cm in women to evaluate reduced muscle mass. However, there was no significant difference in the prevalence of 30-day mortality between the two groups when using CC < 31 cm in both genders as a cutoff point for assessing reduced muscle mass (Table 1).

#### 3.8.1 The ability of GLIM malnutrition diagnostic criteria that use CC ≤ 34 cm in men and CC ≤ 33 cm in women as cutoff points for evaluation of reduced muscle mass in the prediction of clinical outcomes

When considering CC ≤ 34 cm for men and CC ≤ 33 cm for women to evaluate reduced muscle in GLIIM criteria as shown in Table 1, the age, CCI, and medical history of cancer are considered confounding factors. No significant relationship was detected between diagnosed malnutrition and hospital mortality in any of the defined models. In the crude model, malnutrition significantly increased the chance of 30-day hospital readmission, 30-day hospital mortality, and 60-day mortality by 2.85, 2.98, and 3.13 times, respectively. Meanwhile, malnutrition did not significantly change the risk of in-hospital mortality either before or after the adjustments. After applying adjustments based on CCI (model 1), it was revealed that malnutrition significantly increased the chance of 30-day hospital readmission and 60-day mortality by 2.59 and 2.37 times, respectively. Furthermore, after performing adjustments based on age and medical history of cancer (model 2), it was demonstrated that the chance of prolonged hospital stays, 30-day hospital readmission, and 60-day mortality significantly increased by malnutrition 1.69, 2.10, and 2.70 times, respectively (Table 3).

**Table 3:** Predictive validity of GLIM criteria for malnutrition diagnosis: multivariate analysis.

Using CC ≤ 34 for men and CC ≤ 33 for women to evaluate low muscle mass			Using MUAC < 23 for men and MUAC < 22 for women to evaluate low muscle mass		Using CC < 31 for both genders to evaluate low muscle mass	
Dependent variable	OR^*^/HR^**^ (CI 95%)	*p*-Value	OR^*^/HR^**^ (CI 95%)	*p*-Value	OR^*^/HR^**^ (CI 95%)	*p*-Value
Prolonged LOS (>6 days) ^*^						
Crude	1.34 (0.85, 2.10)	0.20	1.35 (0.87, 2.08)	0.17	1.66 (1.07, 2.56)	**0.02**
Model I	1.44 (0.90, 2.30)	0.12^a^	1.44 (0.92, 2.26)	0.10^a^	1.83 (1.16, 2.87)	**0.009** ^a^
Model II	1.69 (1.05, 2.73)	**0.02** ^b^	1.57 (1.00, 2.47)	**0.04** ^c^	2.13 (1.33, 3.41)	**0.002** ^d^
Hospital mortality^**^						
Crude	3.10 (0.90, 10.68)	0.07	1.73 (0.69, 4.37)	0.24	1.70 (0.65, 4.47)	0.27
Model I	2.90 (0.81, 10.31)	0.09^a^	1.57 (0.62, 4.00)	0.33^a^	1.48 (0.55, 3.95)	0.43^a^
Model II	3.15 (0.87, 11.35)	0.07^b^	1.78 (0.70, 4.53)	0.22^c^	1.58 (0.58, 4.31)	0.37^d^
30-day hospital readmission^*^						
Crude	2.85 (1.58, 5.15)	**<0.001**	1.92 (1.16, 3.19)	**0.01**	1.71 (1.03, 2.84)	**0.03**
Model I	2.59 (1.42, 4.73)	**0.002** ^a^	1.74 (1.04, 2.92)	**0.03** ^a^	1.52 (0.90, 2.56)	0.11^a^
Model II	2.10 (1.09, 4.05)	**0.02** ^b^	1.50 (0.84, 2.66)	0.16^c^	1.37 (0.76, 2.48)	0.28^d^
30-day mortality^*^						
Crude	2.98 (1.20, 7.44)	**0.01**	2.07 (0.99, 4.29)	0.05	2.04 (0.97, 4.28)	0.05
Model I	2.28 (0.89, 5.82)	0.08^a^	1.61 (0.75, 3.43)	0.21^a^	1.52 (0.70, 3.29)	0.28^a^
Model II	2.42 (0.94, 6.20)	0.06^b^	1.99 (0.95, 4.17)	0.06^c^	1.39 (0.63, 3.09)	0.41^d^
60-day mortality^*^						
Crude	3.13 (1.59, 6.13)	**<0.001**	2.66 (1.51, 4.68)	**<0.001**	2.64 (1.48, 4.70)	**<0.001**
Model I	2.37 (1.17, 4.77)	**0.01** ^a^	2.10 (1.16, 3.80)	**0.01** ^a^	1.99 (1.08, 3.64)	**0.02** ^a^
Model II	2.70 (1.35, 5.41)	**0.005** ^b^	2.55 (1.44, 4.52)	**0.001** ^c^	2.06 (1.12, 3.77)	**0.01** ^d^

^*^Logistic regression; ^**^Cox regression.

a Model adjusted for CCI.

b Model adjusted for age and medical history of cancer.

c Model adjusted for medical history of cancer.

d Model adjusted for age, age category, and medical history of cancer and CVA.

CCI, Charlson comorbidity index; CVA, cerebral vascular accident; CC, calf circumference; MUAC, mid-upper arm circumference; GLIM, Global Leadership Initiative on Malnutrition; LOS, length of hospital stays; HR, hazard ratio; OR, odds ratio. Statistical significance with a *p*-value <0.05 is indicated in bold.

#### 3.8.2 The ability of GLIM malnutrition diagnostic criteria that uses MUAC < 23 cm in men and MUAC < 22 cm in women as cutoff points for evaluation of reduced muscle mass in the prediction of clinical outcomes

The CCI and the medical history of cancer were confounding factors when malnutrition was diagnosed by GLIM criteria using MUAC<23 cm in men and MUAC<22 cm in women to assess reduced muscle mass (Table 1). In the crude model (without adjustments) and after applying adjustments based on the CCI (model 1), it was shown that malnutrition significantly increased the chance of 30-day hospital readmission and 60-day mortality (in the crude model: by 1.92 and 2.66 times, respectively, and in model 1 by 1.74 and 2.10, respectively). Furthermore, after executing adjustments based on the medical history of cancer (model 2), it was shown that malnutrition increased the chance of prolonged hospital stay and 60-day mortality by 1.57 and 2.55 times, respectively. In addition, no significant relationship between malnutrition and the risk of hospital mortality or 30 days was detected (Table 3).

#### 3.8.3 The ability of GLIM malnutrition diagnostic criteria that use CC < 31 cm in both gender cutoff points for evaluation of reduced muscle mass in the prediction of hospital clinical outcomes

As shown in Table 1, age, age category (≥65 years), and medical history of cancer and CVA were identified as confounding factors. In the crude model (without adjustments), malnutrition significantly increased the chance of prolonged hospital stays, 30-day hospital readmission, and 60-day mortality by 1.66, 1.71, and 2.64 times, respectively. After applying adjustments based on CCI, it was demonstrated that malnutrition increased the chance of prolonged hospital stays and 60-day mortality by 1.83 and 1.99 times, respectively. In addition, the chance of prolonged hospital stays and 60-day mortality increased significantly by malnutrition after adjustment based on age, age category, history of cancer, and CVA by 2.13 and 2.06 times, respectively. No relationship was detected between malnutrition and the odds of 30-day mortality and the risk of hospital mortality (Table 3).

## 4 Discussion

This validation study showed that GLIM criteria as a malnutrition diagnostic tool based on an anthropometric cutoff point that was chosen to assess reduced muscle mass could have a wide range of accuracy, agreement, distinguishing ability, sensitivity, specificity, and clinical outcomes prediction ability. Although it seems that all three types of GLIM tools that use different cutoff points to evaluate reduced muscle in this current study were almost acceptable compared to the SGA tool as a reference method, each of them was superior to the other in some features. In this regard, using a cutoff point of CC ≤ 34 cm in men and CC ≤ 33 cm in women to evaluate the reduced muscle mass compared to other anthropometric cutoff points investigated in this study had lower accuracy and ability to distinguish malnourished patients and also, its specificity was unsatisfactory, compared to SGA as a reference tool. However, using the other two methods for reduced muscle mass assessment including MUAC <23 cm in men and MUAC <22 cm in women or CC < 31 cm in both genders led to GLIM criteria had good accuracy and malnourished distinguishing ability, and satisfactory sensitivity and specificity compared to SGA as the reference method. In addition, these approaches had substantial agreements with SGA, while when identifying the CC ≤ 34 in men and CC ≤ 33 cm as a reduced muscle mass, these agreements decreased to moderate.

In this study, the use of the CC < 31 cm as a cutoff point to determine reduced muscle mass in both genders led to a more accurate diagnostic performance of GLIM criteria than other approaches. In all three versions of GLIM criteria, malnutrition increased the chance of prolonged hospital stay, 30-day hospital readmission, and 60-day mortality. In the crude model, the GLIM criteria that use CC ≤ 34 in men and CC ≤ 33 cm in women to evaluate the reduced muscle mass could significantly increase the chance of 30-day mortality while after multivariate analysis adjusted for confounders, this relationship disappeared. The reliability of GLIM malnutrition diagnostic criteria in all of the three methods was identified as acceptable.

A study conducted by Maffini et al. aimed to validate the GLIM criteria in hospitalized patients by using two different methods for detecting reduced muscle mass: (a) CC ≤ 34 cm in men and CC ≤ 33 cm in women and (b) A MUAC value lower than the fifth percentile. The results demonstrated that using GLIM criteria had a good accuracy and substantial agreement compared to the SGA as a reference method (31). However, the highest sensitivity and specificity of GLIM criteria compared to the SGA were obtained when calf circumference and MUAC methods were applied, respectively. Furthermore, Maffini et al. reported a significant association between diagnosed malnutrition by GLIM criteria in both approaches for detecting reduced muscle mass with prolonged hospitalization (≥5 days) and in-hospital death (31). In this regard, the study conducted by Beretta et al. reported that malnutrition diagnosed by GLIM criteria with considering CC < 32 cm for women and < 33 cm for men as low muscle mass had a significant association with in-hospital mortality in older surgical patients (32). However, GLIM criteria had no significant relationship with in-hospital death when a MUAC value lower than the fifth percentile was considered reduced muscle mass (32). In the other study conducted by Brito et al. that aimed to validate GLIM criteria in hospitalized patients and applied CC ≤ 34 cm in men and CC ≤ 33 cm in women as a cutoff point for evaluation of reduced muscle mass, the agreement between GLIM criteria and SGA was substantial, GLIM criteria had a good ability for distinguishing malnourished patients, and its sensitivity and specificity were satisfactory compared to SGA tool as the reference tool. However, the accuracy of the diagnostic performance of GLIM criteria was not reported (16). In our study, using this approach for detecting reduced muscle mass led to GLIM criteria having a moderate agreement with SGA, fair ability to distinguish malnourished patients, satisfactory sensitivity, and unsatisfactory specificity. This discrepancy may be due to the fact that Brito et al., in addition to measuring calf circumference, also used the adductor pollicis muscle thickness (APMT) to assess reduced muscle mass (16). Furthermore, these findings could be explained by differences in muscle mass and body composition between the Iranian and Brazilian populations. In our validation study, the predictive validity of GLIM criteria for various hospital clinical outcomes including hospital LOS, prolonged hospital stay (LOS > 6 days), 30-day hospital readmission, and 60-day mortality was confirmed. However, the power of prediction ability was dependent on the method and cutoff that was applied for detecting reduced muscle mass. Furthermore, in the crude model of the Brito et al. study, malnutrition significantly increased the chance of readmission 1.65 times, while after the multivariate analyses, this association disappeared (16). In the study conducted by Contreras-Bolívar et al., malnutrition diagnosed by using GLIM criteria had a significant association with longer hospital stays and 6-month mortality in hospitalized cancer patients (17). However, in some previous studies, the non-significant association between malnutrition detected with GLIM criteria and hospital LOS was reported (16, 18, 19). This mismatch in the results could be explained by the investigations’ incapacity to perform multivariate analysis. Furthermore, in a study by Brito et al., there was a significant association between malnutrition and an increased chance of prolonged hospital stays, death in 6 months, and increased risk of hospital mortality (16), while in our study, the malnutrition that was diagnosed by GLIM criteria (regardless of the type of approach used to measure muscle mass) was not an independent predictor for hospital mortality. This may be attributed to routine screening, nutrition assessment, and nutrition interventions in our research hospitals. However, other post-discharge clinical outcomes including 30-day hospital readmission and 60-day mortality were significantly associated with malnutrition diagnosed by GLIM criteria, which was in line with the results of previous studies (16, 17, 33, 34). This difference could be explained by the variety of methods applied for the assessment of reduced muscle (anthropometrics or DEXA, MRI, or BIA) or differences in chosen inflammatory markers as a supporting measurement such as CRP, interleukin-6, or insulin-like growth factor, and its cutoff point to identify the presence of inflammation.

To the best of our knowledge, this study is the first validation of the GLIM malnutrition diagnostic criteria in non-critical hospitalized patients evaluating the criterion of reduced muscle mass criterion by using three different approaches and comparing them to find the best approach. The prospective design, assessment of content validity, evaluation of the reliability of the GLIM criteria with the internal consistency method, and evaluation of the predictive validity of the GLIM criteria by using various hospital clinical outcomes and post-discharge clinical outcomes based on the guidance that was proposed by the GLIM working group (12) were other strengths of this validation. However, our study includes some limitations: (a) We apply anthropometric approaches to evaluate reduced muscle mass instead of gold-standard methods such as BIA, DEXA, CT, and MRI. (b) The reliability of GLIM criteria in our study was assessed by the method of evaluating internal consistency and calculating the Kudder-Richarson-20 index (Cronbach’s alpha), while the proposed method to testing reliability by the GLIM working group was inter-rater reliability assessing method (12). (c) We were unable to evaluate the potential impact of several cofounder factors on the post-discharge clinical outcome, such as receiving nutritional intervention after discharge, the severity of the disease, and complications. (d) We used 24-h food recalls of patients and energy coefficients of food groups in the exchange list to calculate energy intake. In addition, changes in the dietary intake of patients in the last few weeks were evaluated via self-reporting of patients by comparing with current food intake by using relative-qualitative comparison instead of applying 3-day food recalls (one on weekends and two on non-weekend days) and using Nutritionist 4 software to analyze the food intakes.

## 5 Conclusion

This validation study revealed that using GLIM malnutrition diagnostic criteria in non-critically hospitalized patients could have acceptable content and concurrent validity compared to the SGA tool as the reference tool. In addition, by calculating the Kuder–Richardson index, the reliability of GLIM criteria was approved. Furthermore, the predictive validity of the GLIM criteria was confirmed since it could predict a wide range of clinical outcomes. Nonetheless, the accuracy, sensitivity, specificity, and distinguishing capability of GLIM criteria varied based on the methods used to assess reduced muscle mass. However, it recommends investigating the validation of GLIM criteria with different methods to find the best approach to use this diagnostic tool. In addition, it suggests conducting future studies to identify the reference interval of anthropometric approaches in each population that were associated with reduced muscle mass and malnutrition.

## Data Availability

The original contributions presented in the study are included in the article/Supplementary material, further inquiries can be directed to the corresponding authors.
