# Supplementary material for: Validation of GLIM criteria for hospital malnutrition diagnosis by comparison of three different anthropometric approaches to evaluate reduced muscle mass: a prospective cohort study
Source: Front Nutr. 2024 Dec 5;11:1438158. doi: 10.3389/fnut.2024.1438158 (PMC11655236; doi:10.3389/fnut.2024.1438158)
Supplement: Supplementary file 1 [file Data_Sheet_1.PDF]

Validation of GLIM criteria for hospital malnutrition diagnosis by comparison of three different anthropometric approaches to evaluate reduced muscle mass. A prospective cohort study

Electronic Supplementary Material

Appendix Tables S1-S4.

Appendix Figure S1.

Supplementary Table 1. Malnutrition diagnosis according to GLIM criteria

Phenotypic Criteria	
Weight loss (%)	>5% weight loss within the past 6 months or >10% beyond 6 months<br>• stage 1 if >5% weight loss within the past 6 months or >10% beyond 6 months<br>• stage 2 if >10% within the past 6 months or >20% beyond 6 months
Low body mass index (kg/m^2)	<20 kg/m^2 if <70 years, or <22 kg/m^2 if >70 years<br>• stage 1 if age < 70 and BMI<20 kg/m^2 or if age≥70 and BMI <22kg/m^2<br>• stage 2 if age <70 and BMI<18.5kg/m^2 or age≥70 and BMI <20 kg/m^2
Low muscle mass	Method a) Using CC ≤34 in males and CC ≤33 in females as the cut-off points for reduced muscle mass.<br><br>Method b) Using the MUAC <23 cm in males and MUAC <22 in females as the cut-off points for reduced muscle mass<br><br>Method c) Using the CC <31 for both genders as the cut-off points for reduced muscle mass<br><br>In patients with edema and ascites in the lower extremities, physical examination (based on the principles mentioned in the original manuscript) was used to determine low muscle mass to prevent overestimation.<br><br>Due to the lack of criteria for distinguishing the severity of malnutrition using anthropometrics measurements, this section cannot be used to classify the severity of malnutrition.
Etiologic Criteria	
Reduced food intake or assimilation	The energy intake was estimated by applying the 24-hour recall, and the energy required by the patient was based on the approvals mentioned in the text. The patient's food intake in the last two weeks was estimated with a qualitative question the patient's intake in the past weeks was what percentage of the current intake (100%, 75%, 50%, 25%, or 0%).<br>Symptoms affecting food intake include nausea, vomiting, anorexia, history of gastric bypass, diarrhea, dysphagia, and diseases related to assimilation or absorption (Crohn's, ulcerative colitis, short bowel syndrome, pancreatitis, and gastric cancer).<br><br>50%ER ≥energy intake for 1 week,, or any reduction for >2 weeks
Inflammation	Acute disease/injury or chronic disease-related<br>• If serum CRP levels were available, 5 mg/L < CRP indicated inflammation and disease burden<br><br>• If serum levels of CRP were not available, the presence of inflammation/disease burden was evaluated based on the principles mentioned in the text of the manuscript.

Abbreviations: BMI, body mass index; CC, calf circumference; GI, gastrointestinal; ER, energy requirements; CRP, C-reactive protein;

Supplementary Table 2. Details of the content validity assessment and its scoring based on the expert panel opinions.

GLIM criteria	***CVI_ clarity***	***CVI_ relevancy***	***CVR***	***Final result***
Inflammation	0.81	1.00	0.87	*Confirmed*
Reduced food intake or assimilation	1.00	1.00	1.00	*Confirmed*
Weight loss (%)	1.00	1.00	1.00	*Confirmed*
Low body mass index (kg/m^2)	1.00	1.00	0.75	*Confirmed*
Low muscle mass	1.00	0.93	0.87	*Confirmed*

Abbreviations: CVI, Content Validity Index; CVR, Content Validity Ratio

Supplementary Table 3. Demographic information, nutritional features, and clinical outcomes of hospitalized patients included in this study

Variables		All patients	Males	Females	P Value[*]
Number (%)		332	201 (60.5%)	131 (39.5%)	
Age yr		58 (43, 67.7)	58 (43, 67)	57 (44, 69)	0.87[b]
Age≥65 yr		107 (32.2%)	65 (32.3%)	42 (32.1%)	0.95[a]
PMH	Cancer	86 (25.9%)	58 (28.9%)	28 (21.4%)	0.12[a]
	HTN	52 (15.7%)	24 (11.9%)	28 (21.4%)	**0.02[a]**
	DM	48 (14.5%)	24 (11.9%)	24 (18.3%)	0.10[a]
	Surgery	50 (15.1%)	26 (12.9%)	24 (18.3%)	0.18[a]
	CKD	16 (4.8%)	7 (3.5%)	9 (6.9%)	0.15[a]
	CVA	14 (4.2%)	7 (3.5%)	7 (5.3%)	0.41[a]
	Other PMH	94 (28.3%)	52 (25.9%)	42 (32.1%)	0.22[a]
CCI		3 (1, 4)	2 (1, 4)	3 (1, 4)	0.66[b]
Energy intake (kcal/day)		1004.0 (315.0, 1578.2)	523.5 (0.0, 1113.5)	907.0 (0.0, 1349.0)	0.09[b]
Height		1.68 (1.60, 1.75)	1.70 (1.65, 1.77)	1.62 (1.58, 1.66)	**<0.001[b]**
Malnutrition Using SGA		144 (43.4%)	88 (43.8%)	56 (42.7%)	0.85[a]
Severity of malnutrition Based on SGA	Moderate	75 (22.6%)	41 (20.4%)	34 (26.0%)	
	Severe	69 (20.8%)	47 (23.4%)	22 (16.8%)	
GLIM criteria					
Malnutrition Using GLIM (a)		211 (63.6%)	134 (66.7%)	77 (58.8%)	0.14[a]
Severity of malnutrition Based on GLIM (a)	Moderate	115 (34.6%)	73 (36.3%)	42 (32.1%)	
	Severe	96 (28.9%)	61 (30.3%)	35 (26.7%)	
Malnutrition Using GLIM (b)		153 (46.1%)	91 (45.3%)	62 (47.3%)	0.71[a]
Severity of malnutrition Based on GLIM (b)	Moderate	57 (17.2%)	30 (14.9%)	27 (20.6%)	
	Severe	96 (28.9%)	61 (30.3%)	35 (26.7%)	
Malnutrition Using GLIM (c)		165 (49.7%)	101 (50.2%)	64 (48.9%)	0.80[a]
Malnutrition Using GLIM (c)	Moderate	69 (20.8%)	40 (19.9%)	29 (22.1%)	
	Severe	96 (28.9%)	61 (30.3%)	35 (26.7%)	

Current weight (Kg)	65.1 (55.2, 75.2)	66.0 (56.7, 77.2)	63.5 (53.8, 72.5)	0.05[b]
Usual weight (kg	70.0 (60.0, 80.0)	70.0 (60.0, 80.0)	67.0 (56.0, 75.0)	0.10[b]
Weight loss (%)	134 (40.4%)	80 (39.8%)	54 (41.2%)	0.79[a]
BMI (kg/m^2)	23.6±5.0	23.1±4.8	24.4±5.3	**0.03[c]**
Low BMI	95 (28.6%)	57 (28.4%)	38 (29.0%)	0.89[a]
Calf circumference (cm)	32.0 (29.5, 35.0)	31.5 (29.4, 34.0)	32.0 (29.5, 35.0)	0.27[b]
Mild-arm circumference (cm)	25.6 (23.0, 28.9)	25.4 (23.0, 29.0)	26.0 (22.0, 28.5)	0.14[b]
Reduced muscle mass (a)	237 (71.4%)	153 (76.1%)	84 (64.1%)	**0.01[a]**
Reduced muscle mass (b)	62 (18.7%)	40 (19.9%)	22 (16.8%)	0.47[a]
Reduced muscle mass (c)	126 (38.0%)	79 (39.3%)	47 (35.9%)	0.53[a]
Reduced food intake or assimilation	114 (34.3%)	63 (31.3%)	51 (38.9%)	0.15[a]
Nutrition impact symptoms	150 (45.2%)	82 (40.8%)	68 (51.9%)	**0.04[a]**
Inflammation	225 (67.8%)	139 (69.2%)	86 (65.6%)	0.50[a]
Serum CRP level (mg/L) (N=153)	18.6 (4.4, 104.3)	20.9 (5.5, 105.3)	15.1 (3.7, 104.1)	0.46[b]
LOS	6 (4, 9)	6 (4, 9)	6 (4, 10)	0.55[b]
Prolong hospital stay (>6 days)	158 (47.6%)	93 (46.3%)	65 (49.6%)	0.55[a]
Hospital mortality	20 (6.0%)	12 (6.0%)	8 (6.1%)	0.95[a]
30-days hospital readmission N=326	83 (25.5%)	55 (28.1%)	28 (21.5%)	0.18[a]
30-days mortality N=326	34 (10.4%)	19 (9.7%)	15 (11.5%)	0.59[a]
60-days mortality N=323	65 (20.1%)	33 (17.1%)	32 (24.6%)	0.09[a]

a) Chi-square test.

b) Manne-Whitney test.

c) Student t-test.

d) Fisher Test.

Data is presented as a number (%) as a median (IQR), or as mean ± SD, *) P value for comparison between two males and females

Malnutrition Using GLIM (a) and Reduced muscle mass (a): Applying CC≤34 cm in males and CC≤33 in females as the cut-off point for evaluating the reduced muscle mass.

Malnutrition Using GLIM (b) and Reduced Muscle Mass (b): Applying MUAC<23 cm in males and CC< 22 cm in females as the cut-off point for evaluating the reduced muscle mass.

Malnutrition Using GLIM (c) and Reduced muscle mass (c): Applying CC <31 cm in both genders as the cut-off point for evaluating the reduced muscle mass.

Abbreviations: BMI, body mass index; CC, calf circumference; GI, gastrointestinal; ER, energy requirements; CRP, C-reactive protein; GLIM, Global Leadership Initiative on Malnutrition

Supplementary Table 4. Prevalence of symptoms that affect the nutrition status of hospitalized patients.

Variables	All patients	Males	Females	P Value*
Anorexia	84 (25.3%)	42 (20.9%)	42 (32.1%)	0.02[a]
Nausea and vomiting	41 (12.3%)	24 (11.9%)	17 (13.0%)	0.77[a]
Pain when eating	26 (7.8%)	14 (7.0%)	12 (9.2%)	0.46[a]
Constipation	19 (5.7%)	11 (5.5%)	8 (6.1%)	0.80[a]
Diarrhea	10 (3.0%)	3 (1.5%)	7 (5.3%)	0.04[b]
Dyspnea	5 (1.5%)	4 (2.0%)	1 (0.8%)	0.37[b]
Other symptoms	14 (4.2%)	8 (4.03%)	6 (4.6%)	0.79[a]

a) Chi-square test.
b) Fisher Test.

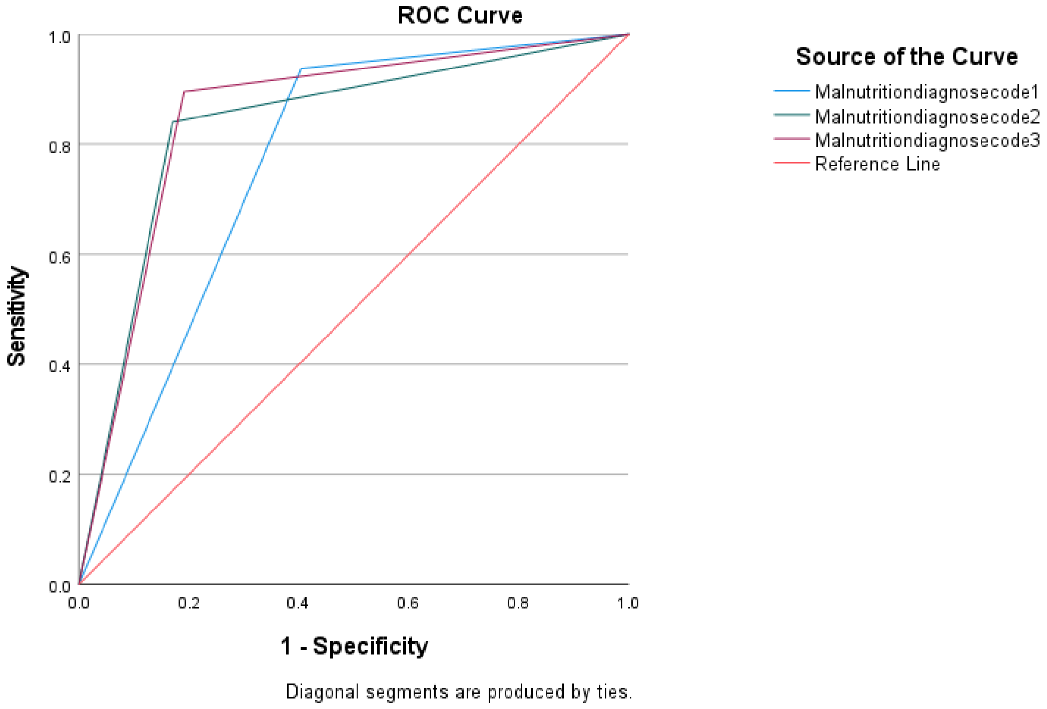

Supplementary Figure 1. Receiver-operating characteristic (ROC) curve plot illustrates the true positive rate (sensitivity) against the false positive rate (one-specificity) at the GLIM criteria against the SGA tool as the reference method.

Malnutrition diagnosis code 1: Indicate applying the GLIM criteria by using the CC ≤34 cm in males and CC ≤ 33 in females as the cut-off point for evaluating the reduced muscle mass.

Malnutrition diagnosis code 2: Indicate applying the GLIM criteria by using the MUAC <23 cm in males and MUAC <22 cm in females as the cut-off point for evaluating the reduced muscle mass.

Malnutrition diagnosis code 3: Indicate applying the GLIM criteria by using the CC <31 cm in both genders as the cut-off point for evaluating the reduced muscle mass.

Reference line: SGA tool
